# Les tumeurs primitives de la glande submandibulaire: à propos de 25 cas

**DOI:** 10.11604/pamj.2015.22.232.7275

**Published:** 2015-11-11

**Authors:** Belhoucha Btissam, Zahra Saidi, Hsaine khaoula, Rochdi Youssef, Aderdour Lahcen, Khouchani Mouna, Raji Abdelaziz

**Affiliations:** 1 Department of ENT, CHU Med VI, Marrakech 2360, Morocco; 2 Department of Oncology and Radiotherapy, CHU Med VI, Marrakech, Morocco

**Keywords:** Glande sous maxillaire, carcinome adénoïde kystique, adénome pléomorphe, chirurgie, radiothérapie, Sub-mandibular gland, adenoide cystic carcinoma, pleomorphic adenoma, surgery, radiotherapy

## Abstract

Les tumeurs des glandes salivaires sont rares, elles constituent 3% des tumeurs de la tête et du cou et 0,6% des tumeurs humaines. La pathologie tumorale de la glande sous-maxillaire est moins fréquente que celle de la parotide et pose autant de problèmes aussi bien diagnostiques que thérapeutiques, surtout l'opportunité de la radiothérapie post-opératoire et l'attitude vis-à-vis des récidives. L'objectif de notre étude est de discuter les problèmes diagnostiques, les résultats thérapeutiques et évolutifs que présentent ces tumeurs afin d'en dégager l'attitude thérapeutique la plus appropriée en se basant sur une étude rétrospectif incluant une série de 25 cas de tumeurs de la glande sous maxillaire colligées aux services d'orl et au service de radiothérapie du CHU MED VI de Marrakech sur une période allant de Janvier 2009 jusqu'au décembre 2014. L’étude a concerné 09 hommes et 18 femmes. La moyenne d’âge était de 48 ans (25 - 71 ans), La tuméfaction sous mandibulaire était le principal symptôme révélateur. Le caractère douloureux a été signalé par 09 patients. Une seule paralysie du rameau mentonnier du nerf facial a été objectivée au moment de l'examen chez un malade. Les adénopathies cervicales ont été retrouvées chez 09 patients. La localisation jugulo-carotidienne haute homolatérale est la plus fréquente. Sur le plan thérapeutique, une sous maxillectomie a été réalisée chez 24 malades dont 02 réopéré pour récidive d'un adénome pléomorphe et un patient pour un carcinome adénoïde kystique. 3 tumeurs malignes n'ont pu bénéficier d'aucun traitement chirurgical du fait du caractère explosif de la lésion Les résultats histopathologiques ont montré une répartition largement dominée par l'adénome pléomorphe pour les tumeurs bénignes (66,66% des tumeurs bénignes), et le carcinome adénoïde kystique pour les tumeurs malignes (37,5% des tumeurs malignes). Neuf malades de notre série ont été irradiés en post opératoire. Deux tumeurs bénignes ayant présenté une première récidive sont toutes des adénomes pléomorphes, 04 tumeurs malignes ont présenté une première récidive (03 carcinome adénoïde kystique, 01 carcinome epidermoide) et un carcinome adénoïde kystique a présenté une deuxième récidive après un complément radiothérapeutique. La pathologie tumorale de la glande sous-maxillaire est complexe, dominée par les tumeurs malignes, elle pose des problèmes diagnostiques et thérapeutiques; Son diagnostic est orienté par des arguments cliniques et radiologiques, repose sur l'analyse anatomopathologique de la pièce d'exérèse opératoire. Un retard diagnostic joint a un traitement initial inadéquat assombrit d'avantage son pronostic.

## Introduction

Les tumeurs des glandes salivaires sont relativement rares, représentant moins de 3% de l'ensemble des tumeurs chez l'homme. La pathologie tumorale de la glande sous-maxillaire est moins fréquente que celle de la parotide et pose autant de problèmes aussi bien diagnostiques que thérapeutiques, surtout l'opportunité de la radiothérapie post - opératoire et l'attitude vis-à-vis des récidives [1–3].

## Méthodes

L'objectif de notre étude est de discuter les problèmes diagnostiques, les résultats thérapeutiques et évolutifs que présentent ces tumeurs afin d'en dégager l'attitude thérapeutique la plus appropriée en se basant sur une étude rétrospectif incluant une série de 25 cas de tumeurs de la glande sous maxillaire colligées aux services d'orl et au service de radiothérapie du CHU MED VI de Marrakech sur une période allant de Janvier 2009 jusqu'au décembre 2015.

## Résultats

**Figure 1:**
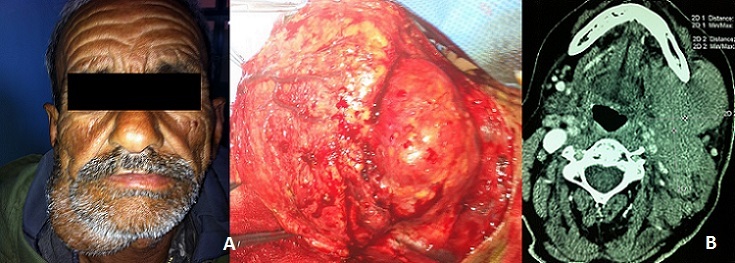
(A) tumeur sous maxillaire droite en pré et per opératoire; (B) processus tumorale de la glande sous maxillaire mesurant 3x4x10cm hétérogène et infiltrant.

**Figure 2:**
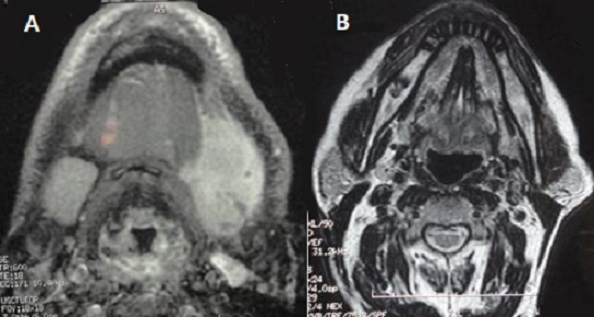
IRM en coupe axial montrant: (A) lymphome de la glande sous maxillaire; (B) rémission complète au contrôle post chimiothérapie.

L’étude a concerné 08 hommes et 17 femmes. La moyenne d’âge était de 48 ans (25 - 71 ans), Le délai moyen d’évolution était relativement court pour les tumeurs malignes (12 mois), voire plus long pour les lésions bénignes (3 ans). La tuméfaction sous mandibulaire était le principal symptôme révélateur, mesurant entre 2 et 6 cm associée à une effraction cutanée dans 02 cas (7%). Le caractère douloureux a été signalé par 09 patients. Une seule paralysie du rameau mentonnier du nerf facial a été objectivée au moment de l'examen chez un malade qui s'est révélé porteur d'un carcinome adénoïde kystique. Trois patients ont présenté des antécédents de chirurgie sous mandibulaire dont deux pour adénomes pléomorphes et un patient pour un carcinome adénoïde kystique. Le délai entre la chirurgie et la récidive tumorale chez ces patients variait entre 1an et 20 ans. Les adénopathies cervicales ont été retrouvées chez 09 patients. La localisation jugulo-carotidienne haute homolatérale est la plus fréquente (Figure 1). L’échographie cervicale a été réalisée chez 11 patients. Elle a orienté vers une tumeur maligne en mettant en évidence une masse hypoéchogène de contours irréguliers dans 05 cas, associée à des adénopathies cervicales homolatérales dans 03 cas. Le scanner de la région submandibulaire en coupes axiales et coronales sans et avec injection de produit de contraste est réalisé chez tous les malades, il a permis de faire le bilan d'extension à l'os, aux muscles, aux tissus adipeux et aux ganglions (Figure 2). L'IRM réalisée dans 11 cas a évoqué une tumeur maligne chez 09 patients devant un processus tissulaire hétérogène mal limité, en hypo signal T2 chez 07 patients et devant une lésion mal limitée, infiltrante à centre nécrosé dans 2cas. Un bilan général à la recherche de métastase a été réalisé soit en pré opératoire quand la malignité était évidente, sinon en post opératoire immédiat ou au cours de l’évolution. Il a permis de déceler 2 cas de métastase pulmonaire dont une était sur un carcinome adénoïde kystique et l'autre sur carcinome épidermoïde. Sur le plan thérapeutique, une sous maxillectomie a été réalisée chez 24 malades dont 02 réopéré pour récidive d'un adénome pléomorphe et un patient pour un carcinome adénoïde kystique. 3 tumeurs malignes n'ont pu bénéficier d'aucun traitement chirurgical du fait du caractère explosif de la lésion. Un curage ganglionnaire a été réalisé devant la présence d'adénopathie palpable (9 curages fonctionnels) sauf pour 04 cas de LMNH confirmé histologiquement. Les résultats histopathologiques ont montré une répartition largement dominée par l'adénome pléomorphe pour les tumeurs bénignes (66,66% des tumeurs bénignes), et le carcinome adénoïde kystique pour les tumeurs malignes (37,5% des tumeurs malignes) (Tableau 1). Neuf malades de notre série ont été irradiés en post opératoire. Trois patients ayant des tumeurs très étendues, jugées inextirpables, ont bénéficié d'une radio-chimiothérapie. Les cas ayant un LMNH ont bénéficié d'une chimiothérapie suivie d'une radiotherapie à 30gy.FIG3 Sur le plan évolutif, le recul moyen dans notre série était de 33 mois avec des extrêmes allant de 7 mois à 60 mois. Durant ce délai, deux tumeurs bénignes ayant présenté une première récidive sont toutes des adénomes pléomorphes, 04 tumeurs malignes ont présenté une première récidive (03 carcinome adenoide kystique,01 carcinome epidermoide) et un carcinome adenoide kystique a présenté une deuxième récidive apres un complément radio thérapeutique. Le traitement proposé pour ces récidives a toujours été chirurgical sauf pour la tumeur maligne qui a présenté une deuxième récidive; une chimiothérapie a alors été réalisée. Par ailleurs 04 malades sont décédés durant la première année d’évolution de leurs maladies (03 carcinome adenoide kystique, 01 carcinome epidermoide), 02 autres ont été perdus de vue dans des délais variables de suivi et 19 patients n'ont pas présenté de récidive ni métastase.

**Tableau 1:** Répartition histopathologique des lésions selon la classification histologique de l'OMS de 1991 (1) K = carcinome

Tumeurs bénignes	Nombre	Tumeurs malignes	Nombre
Adénome pléomorphe	06 cas	k. adénoïde kystique	06 cas
Oncocytome	01 cas	k. épidermoïde	03 cas
lymphongiome kystique	01cas	adénocarcinome	01cas
fibrome	01 cas	k. muco-épidermoïde	02 cas
		lymphomes MNH	04 cas
Total	09 cas		16cas

## Discussion

Les tumeurs des glandes salivaires sont rares, elles constituent 3% des tumeurs de la tête et du cou et 0,6% des tumeurs humaines [4, 5]. Environ 10% de toutes les tumeurs des glandes salivaires sont localisés dans la glande sous-maxillaire, avec un taux élevé de tumeurs malignes. L′âge moyen de découverte des tumeurs submandibulaires est entre la 4e et la 5e décade. Le sexe ratio global varie selon les études avec une légère prédominance féminine, concordent avec celles de notre étude [1, 2, 5, 6]. Les tumeurs de la glande sous maxillaire se présentent généralement sous forme d'une tuméfaction indolore de croissance lente. La malignité est à suspecter devant une tuméfaction sous mandibulaire dure, douloureuse, et plus ou moins fixe au plan superficiel et ou profond. La présence d'adénopathie satellite palpable ainsi qu'une atteinte cutanée ou osseuse ou nerveuse doivent faire évoquer une éventuelle extension loco-régionale. Cependant aucun de ces signes n'est absolu et la distinction entre bénin et malin est loin d’être schématique [6, 7]. L'intérêt de l'imagerie de la glande sous maxillaire est de procurer au chirurgien une information anatomique ainsi qu'une information sur la nature de la tumeur investiguée, mais aucune n’étant spécifique. Certains auteurs défendent la valeur de l’échographie surtout dans la différentiation entre tumeur bénigne ou maligne [3]. Les images évoquant la malignité sont surtout hétérogènes mal limitées plus ou moins étendues en dehors de la glande avec parfois des adénopathies associées [7]. Dans notre étude l’échographie a orienté vers une tumeur maligne en mettant en évidence une masse hypoéchogène de contours irréguliers dans 05 cas, associée à des adénopathies cervicales homolatérales dans 03 cas. D'autres auteurs jugent que le scanner reste la méthode de choix surtout dans la détermination du bilan d'extension loco-régionale vu sa disponibilité et la richesse des renseignements qu'il offre [8]. L'IRM par ses possibilités d'exploration des tissus mous dans les trois dimensions permet une analyse topographique précise des rapports entre tumeur et axes vasculaires et nerveux, elle présente un intérêt certain dans la visualisation des récidives [3, 8].

Dans cet étude, on a évoqué une tumeur maligne chez 09 patients devant un processus tissulaire hétérogène mal limité, en hypo signal T2 chez 07 patients et devant une lésion mal limitée, infiltrante à centre nécrosé dans 2 cas. Seule l'analyse anatomopathologique de la pièce d'exérèse opératoire est en mesure d'affirmer le diagnostic de nature de la tumeur. L'adénome pléomorphe est la plus fréquente des tumeurs bénignes (47.2%) [1]. Les autres tumeurs bénignes telles que les myoepiteliomes, les adénomes a cellules basales, les oncocytomes ou les kystes sont beaucoup plus rares et n'ont pas de véritable spécificité clinique [1, 4]. La plupart des auteurs s'accordent pour reconnaître que la malignité est plus fréquente sur les lésions sous mandibulaires que parotidiennes; les taux publiés varient de 40% à 55% [1, 3, 4]. Le carcinome adénoïde kystique est la tumeur maligne la plus fréquente de la glande sous maxillaire; il représente 40% [4], suivi par le carcinome mucoépidermoïde (10%), puis le Carcinome épidermoïde (10%), Les autres tumeurs malignes telles que l'adénocarcinome, Carcinome indifférencié et le Carcinome à cellules acineuses sont beaucoup plus rares [1, 3, 4]. Le lymphome, étant une tumeur non épithéliale, représente 20% des tumeurs malignes de la glande sous maxillaire. Ces données rejoignent les résultats retrouvés dans notre série [1, 4].

Le traitement des tumeurs sous maxillaire suscite depuis de nombreuses années des controverses quand au type d'exérèse lésionnelle, l'attitude vis-à-vis des aires ganglionnaires et l′utilité de la radiothérapie complémentaire pour les tumeurs malignes. Le but pour toute chirurgie est d'assurer une exérèse large pour diminuer le taux des récidives et en même temps respecter les structures nerveuses [2, 3]. Le grand principe de la chirurgie des tumeurs bénignes, en particulier l'adénome pléomorphe, est d'en faire une sous maxillectomie par voie externe ce qui permet mettre à l'abri des risques de récidive et de dégénérescence. Devant une tumeur maligne primitive, une sous maxillectomie et un curage ganglionnaire, doivent être effectué [9], vu l'incidence élevée de l'atteinte histologiquement prouvée des ADP cervicales (53%) [1, 4, 9]. Les complications de la sous maxillectomie sont dominées par l'atteinte du rameau mentonnier du nerf facial (7,7%), l'atteinte du nerf grand hypoglosse (2,9%) et l'atteinte du lingual (1,4%) [1, 3, 9]. L'atteinte du rameau mentonnier du nerf facial a été notée chez 03 patients dans notre étude. La radiothérapie post-opératoire a suscité l'intérêt de la plupart des auteurs avec une divergence quant à son mode d'application et à sa technique. Certains auteurs confirment son utilité dans le contrôle loco régional de la maladie, mais ne sont pas parvenus a prouver son efficacité en matière de survie [3, 9]. La dose préconisée est de 54 à 60 gy en post opératoire prophylactique et 66 à 70 gy si tumeur ou adénopathie en place ou limite de résection tumorale à raison de 2 GY par séance, 5 séances par semaine. Les tumeurs non réséquables pour raison médicale ou chirurgicale relèvent d'un traitement par radiothérapie première [3, 9]. La chimiothérapie semble être réservée, du moins aux tumeurs réputées chimio-sensibles (lymphomes et sarcomes) [3, 9, 10]. Quatre patients de notre série, ayant un LMNH; diagnostiqué sur pièce opératoire; ont bénéficié d'une chimiothérapie suivie d'une radiotherapie à 30 gy. Les facteurs pronostiques pour définir les situations cliniques et les stratégies thérapeutiques sont le stade clinique et la taille tumorale, le grade histologique, l'envahissement perinerveux, la qualité de l'exérèse chirurgicale et la réalisation ou non d'une radiothérapie postopératoire [4, 10]. Une surveillance à long terme basée sur l'examen clinique et l'imagerie, s'impose afin de dépister les récidives et les métastases.

## Conclusion

La pathologie tumorale de la glande sous-maxillaire est complexe, dominée par les tumeurs malignes, elle pose des problèmes diagnostiques et thérapeutiques; Son diagnostic est orienté par des arguments cliniques et radiologiques, repose sur l'analyse anatomopathologique de la pièce d'exérèse opératoire. Un retard diagnostic joint a un traitement initial inadéquat assombrit d'avantage son pronostic.
